# Changes in Accommodative and Binocular Function following Phakic Intraocular Lens for High and Low-to-Moderate Myopia

**DOI:** 10.3390/ijerph19116716

**Published:** 2022-05-31

**Authors:** Esther López-Artero, Francisco Poyales, Nuria Garzón, Alicia Matamoros, Alba Sáez, Ying Zhou, María García-Montero

**Affiliations:** 1 Miranza Group, C/Galileo 104, 28003 Madrid, Spain; estherl.artero1984@gmail.com (E.L.-A.); ioamadrid@hotmail.com (F.P.); mgarzonj@ucm.es (N.G.); amatamoros@ioamadrid.com (A.M.); asaez@ioamadrid.com (A.S.); 2 Optometry and Vision Department, Faculty of Optics and Optometry, Complutense University of Madrid, 28037 Madrid, Spain; 3 OCULUS Iberia, S.L., Tres Cantos, 28760 Madrid, Spain; yingzhou@oculus.es

**Keywords:** refractive surgery, phakic intraocular lens, implantable collamer lens (ICL), accommodation, binocular vision, high-myopia and low-to-moderate myopia

## Abstract

The aim was to evaluate accommodative and binocular function of phakic intraocular lens implantable collamer lens (ICL) in high and low-to-moderate myopia. Prospective comparative cohort study with 38 myopic patients who underwent ICL implantation were divided into two groups of 19 patients, each one based on the spherical equivalent (SE): high-power (SE ≤ −6 D) and low-to-moderate (SE > −6 D). The push-up amplitude of accommodation (AA), monocular accommodative facility (MAF), distance and near ocular deviation, near convergence amplitude, near point convergence (NPC), stereopsis, and accommodative convergence/accommodation (AC/A) ratio were assessed before surgery and 1 week and 1 month postoperatively. The mean residual refractive error at 1 month after surgery improved in both groups, 0.18 ± 0.34 D and 0.09 ± 0.26 D, respectively (*p* < 0.001). There was a significant decrease in AA in both groups between preoperatively and at 1-week (*p* = 0.001; *p* = 0.008, respectively) and 1-month follow-up (*p* = 0.001; *p* = 0.008). For the rest of the binocular measurements, no statistically significant postoperative changes were found in any group. This finding suggests follow-up studies on amplitude of accommodation in phakic intraocular lens ICL implantation.

## 1. Introduction

There is a variety of refractive procedures to correct myopia, such as corneal refractive surgery, crystalline-lens replacement with an intraocular lens (IOL), or phakic intraocular lens (P-IOL) implantation. One of the most widely-used and worldwide-used p-IOL types is the implantable collamer lens (ICL; STAAR Surgical Inc., Monrovia, CA, USA), and the outcomes reported in a recent review for myopia correction support its use, confirming the safety, efficacy, and predictability of the procedure [1,2,3].

In any case, although there are many publications showing the outcomes obtained with p-IOL ICL, the references are very limited regarding the evaluation of accommodative and binocular function after the implantation in combination [1,4,5].

It is important to emphasize that myopic patients tend to accommodate less than emmetropes for all target distances when wearing distance corrective lenses [6], and p-IOL implantation in young myopic adults who wish to achieve independence from glasses or contact lenses does not require the extraction of the lens; therefore, the patient’s accommodation is preserved. Furthermore, the new refractive status gives a magnification of image size; however, patients require a much higher change in crystalline lens refraction in contrast to a spectacle-corrected one [7].

Consequently, the accommodative and binocular vision evaluation could be relevant because the refractive change is very high in most of the patients, and there can be a high anisometropia due to the delay between the implantation of the first and the second lens that could decompensated their binocular vision. There is no evaluation comparing the accommodative and binocular changes between patients with different degrees of myopia implanted with these lenses.

Therefore, the aim of this study is to analyze the changes and effect of bilateral implantation of p-IOL ICL on accommodative and binocular function for high and low-to-moderate myopia.

## 2. Materials and Methods

### 2.1. Patients

This prospective and controlled study was conducted at Miranza IOA Clinic Madrid. It included 38 myopic patients who underwent Visian^®^ p-IOL ICL™ (STAAR Surgical Company, Monrovia, CA, USA) implantation by an expert surgeon (FP) from February 2019 to December 2020. They were divided into two groups based on baseline spherical equivalent (SE) obtained after subjective refraction: the high-power group (SE ≤ −6 D) and low-to-moderate power group (SE > −6 D) [8].

The sample size was exclusively calculated based on amplitude of accommodation at one month after surgery, which is the main variable of the study, to detect a difference equal or greater than 2 D, assuming a standard deviation of 2.72 according to the results related to the push-up AA at one month after surgery published by Kamiya et al. [9] accepting an alpha risk of 0.05 and a beta risk of 0.2.

Inclusion criteria were myopia from −0.75 to −20 D and regular astigmatism lower than 1.50 D, age from 18 to 40 years, corrected distance visual acuity (CDVA) better than 20/25, and patients who did not wear contact lenses during the six previous months prior to recruitment. Exclusion criteria were presence of manifest tropia, a history of strabismus surgery, anisometropia greater than 1.5 D, accommodative dysfunction, previous strabismus or intraocular surgery, absence of binocular vision, anterior segment pathologic conditions, and systemic pathologies such as neurological or vascular diseases or pharmacological treatment (antihistamines, bronchodilators, anti-infectives, antiepileptics, analgesics) with side effects on accommodative function.

All patients provided written informed consent. The study was approved by the local ethics committee, and it was performed in accordance with the Declaration of Helsinki. The study was approved by the ethic committee Clínico San Carlos Hospital, Madrid, Spain (CI. 18/493-E).

### 2.2. Surgical Procedure

For surgery with the Visian^®^ p-IOL ICL™ (STAAR Surgical Company, Monrovia, CA, USA), the pupil was dilated 30 min before surgery. After injecting 1% sodium hyaluronate into the anterior chamber, the ICL was implanted in the posterior chamber through a 2.8 mm temporal corneal incision. The following step was to wash away the viscoelastic surgical agent using a balanced salt solution, and a miotic agent (acetylcholine) was then instilled. Postoperative medications included topical antibiotics and steroids. All the surgeries were performed without complications.

For the calculation of the lens power required for each eye, the data taken into account were the patient’s subjective refraction, the anterior chamber depth, keratometry, the white-to-white distance, and the corneal pachymetry measurements, which were performed by corneal tomography Pentacam HR^®^ (Oculus, Wetzlar, Germany) and swept-source IOL Master^®^ 700 biometer. The size of the ICL was calculated with the calculator developed by the manufacturer (https://ocos.staarag.ch/landing/ accessed on 3 February 2020). The target refraction was based on emmetropia.

### 2.3. Visual Assessment Protocol and Accommodative and Binocular Vision Examination

All patients underwent a complete ophthalmological examination before surgery, including a detailed anamnesis, uncorrected distance visual acuity (UDVA) and CDVA measured with a Snellen chart, manifest and cycloplegic refraction (three drops of tropicamide 1% instilled every 5 min and performing the refraction 30 min after the last instillation), slit lamp biomicroscopy, and fundus examination. In addition, corneal topography and biometric measurements were performed with the Pentacam HR^®^.

#### 2.3.1. Accommodative Assessment

Accommodative function was assessed by measuring the monocular amplitude of accommodation (AA) with the push-up method [10] and the monocular accommodative facility (MAF) with a ±2.00 D diagnostic flipper set [11]. To estimate the AA, the subject was asked to fixate on a detailed test object approaching the eye, and when the first slight but sustained blurred image was reported, the test was stopped, obtaining the near point of accommodation. The distance in meters between this point and the eye was converted to its reciprocal to have the AA value in diopters. On the other hand, MAF was evaluated with ±2.00 D flipper lenses at 40 cm (near VA 20/30 letters) over a 1 min period. The plus side of the flipper (+2.00 D) was always presented first. The subjects were instructed to look at the letters and try to keep them clear. When the subject indicated clarity, the ±2.00 D diagnostic flipper was flipped over, and then, the instructions were the same but with the negative side of the flipper (−2.00 D). The loop was repeated, and the MAF value was the number of flipping times within 1 min expressed in cycles per minute (cpm).

#### 2.3.2. Binocular Vision Assessment 

Binocular vision evaluation was based on the results of extraocular motility testing, ocular deviation for distance and near fixation, fusional vergence for near fixation, near point of convergence (NPC), stereopsis, and accommodative convergence/accommodation (AC/A) ratio. In addition, the convergence insufficiency symptom survey (CISS-V15) was assessed according to the authors methodology to quantify associated symptomatology [12,13]. 

Ocular deviation was measured with a prism and alternate cover test (PACT) at distance and near fixation [14]. Fusional vergences were measured for near fixation (40 cm) using an accommodative target (single 20/30 letter) and a horizontal prism bar held in the frontal plane position parallel to the face, and employing the step method, base-out prism was gradually increased to get convergence amplitude. Subjects were instructed to concentrate on the target and to keep it single, to report when it became double (break point), and if they could make the target single again (recovery point). The NPC was assessed as Scheiman et al. previously described with an accommodative target (single 20/30 letter), which was moved slowly toward the patients [15]. The blur, break, and recovery values were recorded in centimeters. These measurements were performed three times, and then, the mean value was used for analysis. Stereopsis was measured at a distance of 40 cm using the Titmus Stereotest (Stereo Optical Inc., Chicago, IL, USA). AC/A ratio was calculated according to the calculated method comparing the near and distance ocular deviation [16]. 

Postoperative visits were performed at 1 week and 1 month after surgery. Follow-up examinations included a full ophthalmological examination carried out by the surgeon and accommodative and binocular assessment performed by trained optometrists.

Accommodative and binocular tests were performed with the best manifest refraction placed in trial frames. 

### 2.4. Data Analysis and Statistics

The main focus of the study was to evaluate binocular vision; therefore, all binocular variables of all patients were included for the analysis. For the monocular variables, only data from the right eyes were presented [17,18]. For binocular vision testing, to measure the ocular deviation at distance and near vision, a positive-sign value represents an esophoria, and a negative-sign value implies exophoria. The statistical analysis was aimed at assessing how binocular vision outcomes had changed as a result of surgical procedure. All the metrics were collected and recorded in a Microsoft Excel worksheet (v. 8, Microsoft Corporation, Redmond, WA, USA) and later exported to an SPSS database (v. 22.0, SPSS Inc., Chicago, IL, USA). Each variable’s descriptive-statistics analysis included mean and standard deviation. As for qualitative metrics, percentage values and number of subjects were calculated. For inferential statistics analysis, Shapiro–Wilks test aiming at testing for normality was performed. Each variable’s data were later evaluated by means of non-parametric (Student’s *t*-test) or parametric (Wilcoxon signed-rank) tests. The *p*-value threshold for statistical significance was set at 0.05.

## 3. Results

The study enrolled 38 patients, and the mean age was 29.8 ± 4.7 years (range 21 to 38 years). The high-power group included 19 patients (53.3% men and 46.7% women), and the mean age was 30.0 ± 5.1 years, and the low-to-moderate-power group included 19 patients (47.4 % men and 52.6% women), and the mean age was 29.6 ± 4.4 years (range 22 to 36 years) There were no differences in age between groups (*p* > 0.05). Demographic data are summarized in Table 1. The mean residual refractive error was 0.16 ± 0.30 D (*p* < 0.001) in the high-power group and 0.03 ± 0.12 D (*p* < 0.001) in the low-to-moderate group one week postoperatively; 0.18 ± 0.34 D (*p* < 0.001) and 0.09 ± 0.26 D (*p* < 0.001), respectively, at one month. All postoperative refractive error values were statistically significant different from preoperative values (*p* < 0.05). 

### 3.1. Accommodative Outcomes

The preoperative AA was 11.38 ± 2.82 D (range 7.40 to 18.18) and 10.70 ± 2.16 D (range 7,69 to 16,67) in the high-power group and in the low-to-moderate group, respectively There was a significant decrease in AA in both groups between preoperatively and one-week follow-up (*p* = 0.001; *p* = 0.008, respectively) and one-month postoperative visit (*p* = 0.001; *p* = 0.008, respectively) (Table 2). Instead, preoperative MAF was 11.8 ± 3.8 cpm (range 6 to 18) and 12.2 ± 2.8 D (range 8 to 17) in the high-power group and in the low-to-moderate group, respectively. MAF increased, but there were statistically significant differences after surgery in neither group (Table 2).

### 3.2. Binocular Vision Outcomes

The extraocular motility examination showed no cases of vertical deviation or decompensated phoria in any of the groups. 

Table 3 shows the distance and near ocular deviation, near convergence amplitude (break and recovery points), and AC/A ratio. There were no statistically significant postoperative changes in all distances evaluated, and the postoperative changes were not statistically significant between groups. 

The mean preoperative stereopsis values (seconds of arc) and one-month postoperative was 41.00 ± 24.93 and 31.19 ± 12.29, respectively, in the high-power group and 41.26 ± 24.25 and 33.97 ± 13.93, respectively, in the low-to-moderate group. There were no statistically significant differences between the visits in both groups.

Regarding NPC (Table 3), there were statistically significant differences in NPC blur in both groups between preoperatively and one-week (*p* < 0.001; *p* = 0.001, respectively) and one-month postoperative visits (*p* < 0.001; *p* = 0.002, respectively). The NPC break and NPC recovery in the high-power group showed a statistically significant increase one week after the surgery (*p* = 0.026; *p* = 0.041, respectively); however, both values reverted to pre-op values one month after surgery. The NPC recovery in the low-to-moderate group showed a significant increase one week after surgery (*p* = 0.036) although it went back to normal one month after surgery (Table 4). 

Finally, the mean preoperative score obtained in the CISS-V15 and one-month postoperatively was 8.4 ± 7.9 and 5.2 ± 4.6, respectively, in the high-power group and 5.3 ± 6.8 and 6.1 ± 7.3, respectively, in the low-to-moderate group, showing no statistically significant differences between both groups.

## 4. Discussion

Despite the sudden change in refraction after ICL implantation and the consequent variation of accommodative demand, the bibliographic references regarding accommodation and binocular function in myopic patients implanted with p-IOLs are scarce [4]. The change in vergence with spectacle correction is much lower than the respective value with p-ICL correction, which implies that the refractive change of the crystalline lens necessary to focus on near objects is much higher with a p-ICL implant [7].

Regarding accommodative outcomes, our results showed a decrease in AA in both groups and did not recover until one month after surgery (Table 1). Others authors have studied the changes in AA one month after surgery [9,19,20,21,22]; however, the methods to measure AA were different (accommodometer, minus lens, and push up methods). The changes in AA reported in the current study are in agreement with other studies [9,20], sharing AA methods only with the study of Wan et al. In addition, Wan et al. and Kamiya et al. also studied the result in the medium–long term, observing a transient decrease in AA values at 3 and 6 months with recovery one year after surgery [9,19,20]. Therefore, Wan et al. [20] showed shorter recovery time for the low myopic group. On the other hand, other authors showed an improvement of AA one month after surgery [19,21,22].

The decrease in AA could be due to several reasons. On the one hand, the change in the position of the ametropia-correcting lens (previously in the eyeglasses or contact lens plane and now inside) induces an increase in the accommodative demand [7], and perhaps, this new situation could lead to a depleted accommodative reserve. Another possible reason may be due to a transient dysfunction of the ciliary muscle during the accommodation process due to the fixation of the ICL [23]. Although statistically significant differences have been found in the current study, these differences have no clinical relevance. Changes of approximately 2.00 D in patients younger than 40 years have no impact on the visual function of patients. In addition, a longer follow-up period could be performed to observe long-term changes.

Another test that evaluates the accommodative function is the MAF, which assesses the ability to change focus quickly (far–near). We could expect that this measurement would also be altered after ICL implantation; however, changes have not been shown. Specifically, a slight increase in its value has been observed, meaning a better ability to make far–near focus changes although without statistical significance. On the other hand, other authors [21,22] with the same method as the current study found statistical significance improve in MAF, concluding that MAF could be enhanced and stabilized at 1 month after the surgery although the differences observed were not clinically relevant, and they did not separate high and low myopia. 

Moreover, binocular vision evaluation can be relevant to reinforce the knowledge about binocular changes after p-IOL implantation. The abrupt emmetropization after p-IOL implantation not only changes the accommodative demand but also leads to a sudden, higher convergence demand in near-vision tasks [24]. 

To respond to the main objectives of the study, different binocular variables were analyzed, and we found no changes in any of them except in the NPC blur point.

Distance and near ocular deviation in the high myopia group tends to ortophoria after surgery. Similar results were reported by Kato et al., finding changes in distance and near ocular deviation that were less exophoric than pre-op values [25].

Near convergence amplitude, break and recovery points showed no changes after surgery, and there were no differences between groups at either one-week or one-month visits (Table 1). The tendency for near convergence amplitude was to decrease slightly after surgery, but these changes have not been statistically significant for any group. Similarly, Ryu et al. did not found statistically significant differences, but they reported a small increase in break and recovery points at one month after p-IOL implantation [26]. Differences in the tendency could be explained by the different method used to measure near convergence amplitude (bar prism vs. handheld rotary prism).

AC/A ratio was similar after p-ICL implantation during the first month after surgery in both groups. These results are in line with the outcomes reported by Chen et al. and Luo et al. [21,22]. On the other hand, Ryu et al. revealed an increase of AC/A ratio during the one-month visit, but progressive stabilization was noted during follow-up periods [26]. 

Phakic IOL implantation has demonstrated high optical quality and potential visual acuity improvement in myopic patients due to retinal magnification [27], so this situation is beneficious for the stereoacuity level in non-strabismus or decompensated binocular vision patients. Hence, in the current study, in normal binocular vision subjects, stereopsis outcomes after ICL implantation did not show statistically significant differences, but the values trended to increase in both low-to-moderate and high myopia. Similar results were found by Kato et al. [25]. Other studies showed improvement in stereopsis after p-ICL implantation, including the stereoblind subjects sample [28], anisometric amblyopic patients [29,30], or abnormal preoperative UCVA and CDVA in each implanted eye [31]. 

The NPC is a basic metric to report accommodative and binocular function. In the current study, no differences were found in any NPC values except a deterioration in NPC blur (value increased) in both low-to-moderate and high myopia group at one week and one month after surgery (Table 2). These results regarding NPC blur point are in concordance with the AA decreased observed. Other authors did not evaluate the NPC blur, only NPC break and NPC recovery points, reporting no changes. The results published by Ryu et al. [26] one month after p-IOL implantation did not show changes, and Chen et al. reported an improvement in NPC break [22]. 

Despite changes in AA and NPC blur one month after p-IOL implantation in both myopic groups, there were no differences in symptoms perceived by patients, according to the CISS-V15. 

Due to the relationship between accommodation and convergence, a decrease in accommodation capacity (identified by a decrease in AA) could trigger a decrease in convergence capacity and an increase in near exophoria, leading to a situation that could decompensate the binocular vision of a healthy patient. Our results show a decrease in the accommodation; however, the convergence has not been altered. After p-IOL implantation, patients did not become more exophoric at near distance, and they did not suffer a worsening of the convergence reserves, the level of stereopsis did not decrease but even slightly improved, and the values of the near point of convergence did not worsen.

The current study has some limitations. One of them is the short follow-up time, and the other is the use of a subjective method for the measurement of the accommodative function. It is necessary to move towards objective methods of measuring accommodation.

## 5. Conclusions

In conclusion, p-IOL ICL implantation did not modify baseline accommodative and binocular vision after the surgery in healthy patients with low-to-moderate or high myopia groups except a decrease in the amplitude of accommodation although this change was not clinically relevant due to the age of the patients included in this study. Future studies would be needed to know the impact of this decrease in pre-presbyopes and emerging presbyopes.

## Figures and Tables

**Table 1:** Demographic data.

		Total Group (*n* = 38)	
Age (y) Mean ± SD (range)		29.8 ± 4.7 (21–38)	
		**High-Power Group** **(*n* = 19)**	**Low-to-Moderate-Power Group** **(*n* = 19)**
Age (y) Mean ± SD (range)		30.0 ± 5.1 (21–38)	29.6 ± 4.4 (22–36)
Gender (%)	Men	53.3 %	47.4%
	Women	46.7%	52.6%
Preop SE (D)		−7.82 ± 1.18	−4.57 ± 1.06

Preop SE, preoperative spherical equivalent; y, years; D, diopter.

**Table 2:** Amplitude of accommodation and monocular facility accommodation for high- and low-to-moderate-power group at one week and one month after surgery.

	High-Power Group			Low-to-Moderate-Power Group		
		Postoperative			Postoperative	
Mean ± SD *p*-value	Preoperative	1 Week	1 Month	Preoperative	1 Week	1 Month
AA (D)	11.38 ± 2.82	8.66 ± 2.24 0.001 *	8.39 ± 2.05 0.001 *	10.70 ± 2.16	8.33 ± 1.74 0.008 *	8.83 ± 2.28 0.008 *
MAF (cpm)	11.8 ± 3.8	14.7 ± 2.2 0.154	13.1 ± 4.5 0.312	12.2 ± 2.8	12.3 ± 4.3 0.343	13.2 ± 3.2 0.234

* Statistically significant; AA, amplitude of accommodation; MAF, monocular accommodative facility; cpm, cycles per minute; D, diopter.

**Table 3:** Distance and near ocular deviation, near convergence amplitude, and AC/A ratio for high- and low-to-moderate-power group at one week and one month after surgery.

	High-Power Group			Low-to-Moderate-Power Group		
		Postoperative			Postoperative	
Mean ± SD *p*-value	Preoperative	1 Week	1 Month	Preoperative	1 Week	1 Month
Distance ocular deviation (Δ)	1.2 ± 3.5	0.5 ± 1.3 0.854	0.3 ± 1.9 0.124	1.1 ± 1.9	0.9 ± 1.6 0.705	1.3 ± 4.0 0.904
Near ocular deviation(Δ)	−4.2 ± 8.4	−6.2 ± 8.0 0.726	−2.4 ± 5.7 0.537	0.5 ± 4.2	0.3 ± 2.1 0.854	−0.9 ± 6.2 0.291
Near convergence amplitude (break) (Δ)	33.5 ± 8.2	31.3 ± 9.5 0.344	30.8 ± 11.3 0.502	35.0 ± 9.9	36.2 ± 10.0 0.611	35.3 ± 7.2 0.725
Near convergence amplitude (recovery) (Δ)	30.6 ± 10.7	28.5 ± 11.0 0.484	28.8 ± 12.5 0.789	33.5 ± 8.2	33.1 ± 11.5 0.720	31.4 ± 9.3 0.753
AC/A calculated	8.1 ± 3.1	8.6 ± 3.1 0.472	7.0 ± 2.3 0.141	6.2 ± 1.4	6.3 ± 0.6 0.579	6.9 ± 2.3 0.358

Statistically significant; Minus sign (exophoria); Δ, prismatic diopters; AC/A, amount of convergence measured in prism diopters per unit (Δ) change in accommodation.

**Table 4:** Near point of convergence: blur, break, and recovery values (cm) for high- and low-to-moderate-power group at one week and one month after surgery.

	High-Power Group			Low-to-Moderate-Power Group		
		Postoperative			Postoperative	
Mean ± SD *p*-value	Preoperative	1 Week	1 Month	Preoperative	1 Week	1 Month
NPC blur	7.8 ± 2.5	11.7 ± 2.4 <0.001 *	12.2 ± 3.3 <0.001 *	8.0 ± 2.1	11.7 ± 2.4 0.001 *	11.3 ± 2.8 0.002 *
NPC break	2.9 ± 3.2	6.8 ± 4.8 0.026 *	5.1 ± 5.1 0.073	4.3 ± 3.3	5.6 ± 4.9 0.649	5.9 ± 4.9 0.255
NPC recovery	5.1 ± 5.5	8.7 ± 7.0 0.041 *	7.5 ± 6.9 0.142	6.5 ± 4.6	10.3 ± 5.2 0.036 *	8.9 ± 5.5 0.124

* Statistically significant. NPC, near point of convergence; cm, centimeters.

## Data Availability

The data that support the findings of this study are available on request from the corresponding author (M.G.-M.). The data are not publicly available due to restrictions.
